# Cobalt-containing bioactive glasses reduce human mesenchymal stem cell chondrogenic differentiation despite HIF-1α stabilisation

**DOI:** 10.1016/j.jeurceramsoc.2017.08.001

**Published:** 2018-03

**Authors:** E. Littmann, H. Autefage, A.K. Solanki, C. Kallepitis, J.R. Jones, M. Alini, M. Peroglio, M.M. Stevens

**Affiliations:** a Department of Materials, Imperial College London, London SW7 2AZ, United Kingdom; b Department of Bioengineering, Imperial College London, London SW7 2AZ, United Kingdom; c Institute of Biomedical Engineering, Imperial College London, London SW7 2AZ, United Kingdom; d AO Research Institute Davos, Clavadelerstrasse 8, 7270, Davos, Switzerland

**Keywords:** Bioactive glasses, Cobalt, Mesenchymal stem cells, Chondrogenesis, Hypoxia-inducible factor-1

## Abstract

Bioactive glasses (BGs) are excellent delivery systems for the sustained release of therapeutic ions and have been extensively studied in the context of bone tissue engineering. More recently, due to their osteogenic properties and expanding application to soft tissue repair, BGs have been proposed as promising materials for use at the osteochondral interface. Since hypoxia plays a critical role during cartilage formation, we sought to investigate the influence of BGs releasing the hypoxia-mimicking agent cobalt (CoBGs) on human mesenchymal stem cell (hMSC) chondrogenesis, as a novel approach that may guide future osteochondral scaffold design. The CoBG dissolution products significantly increased the level of hypoxia-inducible factor-1 alpha in hMSCs in a cobalt dose-dependent manner. Continued exposure to the cobalt-containing BG extracts significantly reduced hMSC proliferation and metabolic activity, as well as chondrogenic differentiation. Overall, this study demonstrates that prolonged exposure to cobalt warrants careful consideration for cartilage repair applications.

## 1 Introduction

Bioactive glasses (BGs) offer an interesting carrier system to deliver therapeutic ions due to the relative ease of modifying their composition and their ability to allow a controlled ion release. The glass network structure of BGs partially dissolves when in contact with aqueous buffers, thereby releasing ions into the surroundings [1], [2], [3], [4], [5], [6]. Such BG ionic dissolution products have been shown to influence cell proliferation, metabolic activity and differentiation [1], [3], [4], [5], [6]. Moreover, the incorporation of selected ion species can greatly impact cellular behaviour in a dose-dependent manner, as previously demonstrated for strontium and cobalt [1], [2], [3], [7]. Ionic dissolution products prepared from strontium-containing BGs have been shown to increase metabolic activity of osteoblasts and to decrease TRAP activity of osteoclasts [3]. A whole genome microarray uncovered an up-regulation of the isoprenoid biosynthesis pathway in human mesenchymal stem cells (hMSCs) after treatment with strontium-containing BG ionic dissolution products [1]. This up-regulation induced changes in the composition of the plasma membrane with likely implications on cell signalling events. Recently, ionic dissolution products prepared from cobalt-containing BGs have been demonstrated to induce a hypoxic response in hMSCs [7], [8]. BGs have found broad applications in tissue engineering, both for hard and soft tissue regeneration [9], [10], [11], [12]. Classically, BGs have been used for bone repair purposes due to their strong bonding to bone and osteogenic properties. However, BGs have similarly been identified as suitable substrates for chondrocyte culture and chondrogenic differentiation of MSCs highlighting their potential as delivery system for therapeutic ions that might further promote cartilage repair [13], [14], [15], [16]. Due to that dual action, BGs have been evaluated for the repair of osteochondral defects, at the interface of bone and cartilage. Osteochondral defects present a challenge for the tissue engineering community especially due to the very limited self-repair capacity of cartilage. When 13-93 BG was used as subchondral substrate in a bi-layered scaffold, it enhanced sulphated glycosaminoglycan (sGAG) production by chondrocytes seeded in the agarose layer above [17]. Interestingly, conditioned medium prepared from 13-93 BG increased sGAG synthesis compared to the control medium and the quality of the resulting cartilage-like matrix indicating a pronounced role of bioactive ion dissolution products. To promote the formation of a stable calcified cartilage layer, sintered polylactide-co-glycolide/45S5 BG composite microspheres were combined with agarose hydrogel [18]. Indeed, the incorporation of BG increased both the ALP activity and sGAG synthesis of chondrocytes significantly. Modification of polylactide-based scaffolds with 13-93 BG particles similarly enhanced sGAG production by human adipose tissue-derived MSCs [16].

Considering these promising results, we sought to further investigate the influence of BG ionic dissolution products on the chondrogenic lineage commitment of human bone marrow-derived MSCs. So far, most studies evaluating BGs for osteochondral repair applications focussed on the “classic” BG compositions 45S5 and 13-93. Here, we aimed to enhance the pro-chondrogenic potential of the BGs by incorporating cobalt ions. Cobalt is a well-established hypoxia-mimicking agent due to its ability to activate the hypoxia-inducible factor-1 (HIF-1) pathway independently of the cellular oxygen level [19]. The HIF-1 pathway is the main regulator of the cell’s response to changes in oxygen tension by triggering the expression of more than 100 hypoxia target genes [20]. HIF-1 is a heterodimeric transcription factor consisting of the oxygen tension-regulated HIF-1α subunit and the constitutively expressed HIF-1β subunit [21]. Activation of the HIF-1 pathway depends on the stabilisation of HIF-1α in the cellular cytoplasm. In normoxic conditions HIF-1α is continuously produced and subsequently degraded through the ubiquitin proteasome system. In hypoxic environments HIF-1α can accumulate, translocate to the nucleus and dimerise with HIF-1β to induce the expression of its target genes. Cobalt ions artificially stabilise HIF-1α by blocking the protein’s degradation independent of oxygen levels. Several studies have successfully evaluated the use of reduced oxygen tension to aid chondrogenic differentiation [22], [23], [24], [25]. As cartilage is an avascular tissue, nutrients as well as oxygen must diffuse from the surface of the joint facing the synovial fluid into the cartilage tissue below creating a decreasing oxygen tension gradient from the surface (5% pO_2_) of the articular cartilage layer to the subchondral bone (0.1% pO_2_) [26], [27], [28]. Enhanced chondrogenesis in low oxygen environments is thought to be primarily mediated through HIF-1α by inducing the expression of pro-chondrogenic genes such as *Sox9* [29], [30], [31]. Previously, a few studies have assessed the effects of cobalt on hMSC chondrogenic differentiation [32], [33], [34]. It was shown that administration of 200 μM CoCl_2_ for 24 h induced chondrogenic marker gene expression in hMSCs and pre-treatment of a murine MSC line enhanced subsequent chondrogenic differentiation [34]. Incorporation of Co^2+^ in alginate-based systems supported chondrogenesis of MSCs in a cobalt-concentration dependent manner [32], [33]. Evaluation of hypoxia-mimicking bioceramics for tissue engineering purposes has been mainly directed to bone regeneration applications with a particular focus on the induction of angiogenesis [7], [8], [35], [36], [37]. Collectively it was demonstrated that cobalt-containing bioceramics enhanced osteogenic markers such as ALP activity and promoted VEGF production in several cell types. Previously, we synthesised a series of cobalt-containing BGs with increasing molar cobalt contents (0, 0.5, 1, 2 and 4 mol%) and demonstrated their hypoxia mimicking function in hMSCs [7], [38]. Ionic dissolution products prepared from these glasses successfully increased HIF-1α activity after 8 h of culture and induced the expression of hypoxia target genes such as vascular endothelial growth factor in hMSCs. Based on the knowledge that activation of the HIF-1 pathway can enhance hMSC differentiation towards the chondrogenic lineage, here, the effects of cobalt-doped hypoxia mimicking BGs on hMSC chondrogenesis were evaluated. It was hypothesised that cobalt, together with other bioactive ions released from the glasses, would promote chondrogenic differentiation of hMSCs.

## 2 Materials and methods

### 2.1 Preparation of bioactive glasses

Glass synthesis and particle preparation were performed as previously described [7], [38]. The CoBGs were prepared using SiO_2_ (Prince Minerals), Na_2_CO_3_, P_2_O_5_, CaCO_3_, and CoCO_3_ (all Sigma-Aldrich) as precursor materials. The precursor materials were mixed, melted at 1400 °C in a platinum-gold crucible for 90 min and quenched into deionised water. The resulting glass frit was allowed to dry between 120 and 150 °C overnight before milling in a planetary ball mill at 500 rpm for 5 min. The glass particle size used in this study was <38 μm in diameter (d = 0.9) obtained by sieving. Cobalt was incorporated in the bioactive glass composition 49.46 mol% SiO_2_, (23.08–X mol%) CaO, X mol% CoO, 26.38 mol% Na_2_O, 1.07 mol% P_2_O_5_ by substitution with calcium on a molar basis (where X was 0, 1, 1.5 or 2 mol% cobalt substitution) (Table 1). Increasing amounts of cobalt were incorporated into the composition to identify an optimal cobalt concentration to support chondrogenesis. The bioactive glasses are referred to as 0%CoBG, 1%CoBG, 1.5%CoBG and 2%CoBG according to their molar cobalt content or as CoBGs if all glass compositions are concerned.

**Table 1:** Glass compositions and molar cobalt for calcium substitution (mol%).

Glass	Co^2+^ for Ca^2+^ substitution	SiO_2_	Na_2_O	CaO	CoO	P_2_O_5_
0%CoBG	0	49.46	26.38	23.08	/	1.07
1%CoBG	1	49.46	26.38	22.08	1.00	1.07
1.5%CoBG	1.5	49.46	26.38	21.58	1.50	1.07
2%CoBG	2	49.46	26.38	21.08	2.00	1.07

### 2.2 Preparation of CoBG conditioned medium and ionic content analysis

To obtain CoBG dissolution ion medium, 1.5 mg/mL of glass particles (<38 μm) were incubated in Minimum Essential Medium Eagle alpha modification (α-MEM) (Gibco^®^) or high glucose Dulbecco's Modified Eagle Medium (DMEM) (Gibco^®^) at 37 °C under constant agitation. Both α-MEM and DMEM contained 20 mM HEPES, 1% (v/v) penicillin/streptomycin (all Gibco^®^). After 4 h, the medium was passed through a 0.2 μm pore filter (Sartorius) to remove remaining particles. The media were stored at 4 °C until use. To determine the ionic composition of the CoBG-conditioned α-MEM and DMEM media, medium samples were analysed by inductively coupled plasma optical emission spectrometry (ICP-OES) using an iCAP6000 Series ICP spectrometer (Thermo Scientific). The CoBG-conditioned medium was allowed to equilibrate in a CO_2_ incubator at 37 °C overnight before use.

### 2.3 Human mesenchymal stem cell expansion and pellet culture

For pellet culture, bone marrow-derived human mesenchymal stem cells (hMSCs) were isolated from human bone marrow obtained from the Hospital of Davos, Switzerland after approval by the local ethical commission (KEK-ZH-NR: 2010-0444/0). After bone marrow homogenisation, hMSCs were isolated by Ficoll^®^ gradient centrifugation and adherence to tissue culture plastic as previously described [39]. hMSCs were expanded in α-MEM supplemented with 10% (v/v) FBS (Seraplus, Pan-Biotech), 1 ng/μL bFGF (Fitzgerald), 1% (v/v) penicillin/streptomycin (Gibco^®^).

For chondrogenic differentiation, hMSCs were cultured as pellets for up to 21 days. In brief, passage 3 hMSCs were re-suspended in chondrogenic differentiation medium (high glucose DMEM medium containing 10 ng/ml TGFβ1 (Fitzgerald), 20 mM HEPES, 1% (v/v) ITS + premix (BD), 100 nM dexamethasone (Sigma Aldrich), 50 μg/mL ascorbic acid (Sigma Aldrich), 1% (v/v) penicillin/streptomycin (Gibco^®^), and 1% (v/v) non-essential amino acids (Gibco^®^). Pellets were formed by centrifuging 200,000 cells in 1 mL per aliquot at 400 g for 10 min in 1.5 mL conical microcentrifuge tubes (Eppendorf). After 3 days, the medium was replaced with 1 mL of control or the various CoBG-conditioned media all containing chondrogenic supplements as described above. The pellets were continuously cultured in the 1.5 mL microcentrifuge tubes and the medium was changed every 2–3 days.

For all other experiments, commercially obtained hMSCs at passages 5–6 were used (PromoCell GmbH, Germany and Lonza, UK).

### 2.4 In-Cell western blotting

The HIF-1α protein abundance in hMSCs in the presence of CoBG-conditioned media was measured by In-Cell Western. In brief, cells were seeded on tissue culture plastic at a density of 20,000 cells/cm^2^ and grown until confluency. CoBG-conditioned medium was added for 4 h or 48 h prior to fixation with 3.7% (v/v) paraformaldehyde in PBS (Sigma). Cells were incubated with the primary antibody anti-HIF-1-alpha [mgc3] (Abcam, 1:200) for 1.5 h at room temperature followed by exposure to the anti-mouse IR800-labeled (Li-Cor, 1:400) secondary antibody for 1 h. The obtained intensity values for HIF-1α were normalised to the respective DNA content per well using the far-red fluorescent DNA dye DRAQ5™ (New England Biolabs, 1:2000). The fluorescent signal was collected and quantified using an Odyssey Infrared Imager (Li-Cor Biosciences). The HIF-1α protein expression was determined after 4 h and 48 h (*n* = 4 and *n* = 3 independent experiments using different donors, respectively). Each condition was run in quadruplicates.

### 2.5 MTT assay

MTT assay was performed to assess the effect of CoBGs on hMSC metabolic activity. In brief, 1.5 × 10^4^ cells/cm^2^ of passage 5–6 hMSCs were seeded in 96-well plates. After 24 h the medium was replaced by control (α-MEM) or CoBG-conditioned α-MEM medium containing 10% (v/v) FBS. As an additional control, α-MEM medium was supplemented with 100 μM CoCl_2_. After 24 h, 4 days and 7 days respectively, cell metabolic activity was measured using the MTT (Sigma-Aldrich) assay following manufacturer’s instructions. Absorbance was measured at wavelength 570 nm with a reference wavelength of 620 nm using a microplate reader (SpectraMax M5, Molecular Devices). Cell metabolic activity was determined in 3 independent experiments using different donors. Each condition was run at least in triplicates.

### 2.6 CyQUANT^®^ direct cell proliferation assay

Changes in cell number in the presence of CoBG-conditioned or control medium over time were determined using the CyQUANT^®^ Direct Cell Proliferation Assay (Life Technologies, UK). The assay was performed according to the manufacturer’s instructions. In brief, 5 × 10^3^ cells/cm^2^ of hMSCs were seeded in 96-well plates and cultured in CoBG-conditioned α-MEM medium containing 10% (v/v) FBS for 24 h, 4 and 7 days. Fluorescence was excited at 480 nm and emission was quantified at 535 nm using a microplate reader (SpectraMax M5, Molecular Devices). Cell proliferation was determined in 3 independent experiments using different donors. Each condition was run at least in triplicates.

### 2.7 Filamentous actin staining

To visualise hMSC morphology in the presence of CoBG-conditioned medium, hMSCs were stained for filamentous actin (F-actin). In brief, passage 5 hMSCs were seeded at a density of 4000 cells/cm^2^ on μ-Slide 8 Well Glass Bottom slides (ibidi^®^, UK) and cultured in CoBG-conditioned or control medium. Control medium (α-MEM) supplemented with 100 μM CoCl_2_ was used as positive control. After 1, 4 and 7 days of culture, cells were fixed with 3.7% (v/v) paraformaldehyde in PBS for 20 min. Cells were permeabilised in 0.2% (v/v) Triton-X 100 in PBS for 15 min and blocked with 1% (v/v) goat serum for 30 min. The actin cytoskeleton was stained with phalloidin-Alexa 488 (Life technologies, UK) and the nuclei were stained with DAPI. Imaging with fluorescent microscope (EVOS^®^ FL Cell Imaging System; Life Technologies) was conducted immediately after staining.

### 2.8 Biochemical assays for sGAG/DNA quantification

In brief, hMSC pellets (3 pellets per group, for each experiment) were digested in 0.5 mg/mL proteinase-K (2.5 U/mg, chromozyme assay; Roche) at 56 °C for 16 h. DNA content was determined by spectrofluorometry using Hoechst 33258 dye (Sigma Aldrich) with calf-thymus DNA as standard (Invitrogen). The sGAG content of the digested pellets or in the retained culture media was determined using the 1,9-dimethylmethylene blue (DMMB) (Sigma Aldrich) dye calorimetric assay with chondroitin-6-sulphate as a standard. All measurements were performed on a 1420 Multilabel Counter Victor 3 (Perkin Elmer). DNA content and sGAG accumulation on day 0 was measured for 3 donors in independent experiments (in triplicates). For the day 21 time point, 5 independent experiments with different donors were performed.

### 2.9 Histology

At day 0 and after 21 days, hMSC pellets were fixed in 70% methanol and incubated in 5% (w/v) sucrose in PBS solution overnight at 4 °C prior to embedding in Jung Tissue Freezing Medium (Leica Biosystems). Sections (10 μm) of pellets were prepared and subjected to Toluidine Blue and Safranin O/Fast Green staining. For each donor, 1 pellet per condition was subjected to histology. Representative sections are shown.

### 2.10 Confocal raman spectral imaging of hMSC pellet sections

After 21 days, hMSC pellets were fixed in 3.7% (v/v) PFA in PBS for 20 min and embedded in Jung Tissue Freezing Medium (Leica Biosystems) prior to being snap frozen. For Raman analysis, 10 μm sections of hMSC pellets were prepared and collected on magnesium fluoride (MgF_2_) slides (Global Optics). Prior to Raman spectral imaging, remaining Jung Tissue Freezing Medium was removed by dH_2_O. Raman spectral imaging was applied to one section per condition of pellets from one donor. Imaging was performed on a confocal Raman microscope (alpha300R+, WITec, Ulm, Germany). A 532 nm laser was used with a 10 × objective; scattered light was directed through a 100 μm fibre. Spectra were collected with a 10 μm step size and 0.3 s integration time. For multivariate data analysis, each Raman spectral image was processed using in-house written analytical methods through Matlab software (2016, MathWorks). Each Raman spectral image was first pre-processed by undergoing a third order polynomial baseline correction, Principal Component Analysis (PCA) assisted cosmic rays removal/outlier and Savitzky–Golay smoothing. K-means clustering was used to segment the pellet regions removing the surrounding background area. Multivariate Curve Resolution (MCR) was used to identify the spectral signatures dominating the data variation (*i.e.* collagen, GAG and lipid bodies). Afterwards, spectra of pure collagen, GAG and lipids were obtained and a non-negative least-squares curve fitting analysis was used to calculate abundance values for these components in each pixel of the Raman images. Using the abundance values a semi-quantification and comparison between the different culture conditions was possible.

### 2.11 Statistical analysis

To assess statistical significance, one-way ANOVA with Tukey *post hoc* test or two-way ANOVA with Bonferroni *post hoc* test were used. *P*-values lower than 0.05 were deemed significant.

## 3 Results

### 3.1 Ionic composition of CoBG-conditioned medium

BG ionic dissolution products have been identified as versatile tools to guide cell behaviour. Here, we sought to study if there was an optimum Co^2+^ concentration range or threshold to direct hMSC chondrogenesis. Increasing amounts of cobalt ions were incorporated into the BG composition 49.46 mol% SiO_2_, (23.08–X mol%) CaO, X mol% CoO, 26.38 mol% Na_2_O, 1.07 mol% P_2_O_5_ by substitution with calcium on a molar basis (where X was 0, 1, 1.5 or 2 mol% cobalt substitution) to create a series of cobalt-doped BGs (Table 1) [7], [38]. CoBG-conditioned medium was prepared by incubating CoBG particles in cell growth medium for 4 h to evaluate the influence of CoBG dissolution products on hMSC chondrogenic differentiation. Incubation of CoBG particles in α-MEM and DMEM resulted in similar dissolution profiles for all ions (Table 2 and Supplement 1). The cobalt ion release into both α-MEM and DMEM increased according to its molar content in the glasses. Co^2+^ concentrations of 117 μM, 174 μM and 249 μM were measured in DMEM medium conditioned with 1%CoBG, 1.5%CoBG and 2%CoBG particles, respectively. These values are within the range of concentrations widely used in cell culture experiments to mimic hypoxia (100–300 μM) [34], [40]. The concentrations of silicon, calcium and phosphorous in the conditioned medium were similar between all glass compositions.

**Table 2:** Tailoring of glass compositions allows controlled release of cobalt ions into DMEM medium. Elemental concentrations of CoBG ionic dissolution products (Si, Ca, P and Co) in DMEM high glucose medium. CoBG particles were incubated in DMEM for 4 h under constant agitation. Ion release was measured by ICP-OES. Elemental concentrations are expressed as mean ± standard deviation (SD) of 4 independent experiments (in μg/mL unless otherwise indicated). ND = non-detectable.

	Si	Ca	P	Co	Co(μM)
DMEM	ND	66 ± 6	29 ± 3	ND	/
0%CoBG	65 ± 8	142 ± 22	14 ± 2	ND	/
1%CoBG	65 ± 6	142 ± 22	17 ± 6	7 ± 1	117 ± 6
1.5%CoBG	66 ± 9	145 ± 16	19 ± 3	10 ± 1	174 ± 11
2%CoBG	66 ± 6	142 ± 22	16 ± 5	15 ± 1	249 ± 15

### 3.2 Effect of CoBG dissolution products on HIF-1α stabilisation

**Fig. 1:**
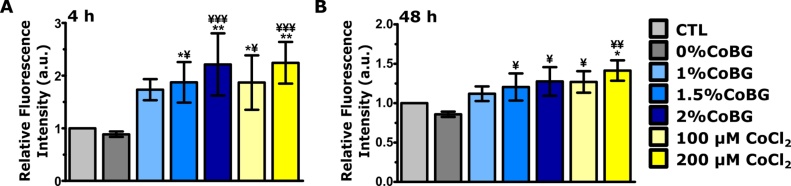
Increased HIF-1α protein abundance in the presence of CoBG. hMSCs were cultured for A) 4 h or B) 48 h in control (CTL), CoCl_2_ or CoBG-conditioned medium. The protein abundance of HIF-1α was quantified by In-Cell Western and normalised to DNA. Data are expressed as mean ± SD of A) *n* = 4 and B) *n* = 3 independent experiments using different donors. Statistical significance was assessed by one-way ANOVA with Tukey *post hoc* test. *p < 0.05 (versus (vs.) control), **p < 0.01, ¥*p *< 0.05 (vs. 0%CoBG), ¥¥*p* *< *0.01, ¥¥¥*p* < 0.001.

To assess whether the CoBG dissolution products mimicked hypoxia in hMSCs, the amount of HIF-1α protein was quantified after 4 h and 48 h of culture in CoBG-conditioned medium (Fig. 1). After 4 h, exposure to 1.5%CoBG, 2%CoBG as well as the 100 μM and 200 μM CoCl_2_ positive control conditions led to a significant increase in the amount of HIF-1α protein compared to control medium and 0%CoBG (Fig. 1A). HIF-1α abundance appeared correlated to the concentration of cobalt within the CoBG dissolution products, with the protein stabilised to a similar degree between 1.5%CoBG and 100 μM CoCl_2_ (as well as between 2%CoBG and 200 μM CoCl_2_). After 48 h of exposure to CoBG-conditioned medium, HIF-1α abundance remained significantly higher than 0%CoBG, suggesting an effect mediated by the incorporation of cobalt within BG (Fig. 1B).

### 3.3 Influence of CoBG dissolution products on hMSC metabolic activity and proliferation

**Fig. 2:**
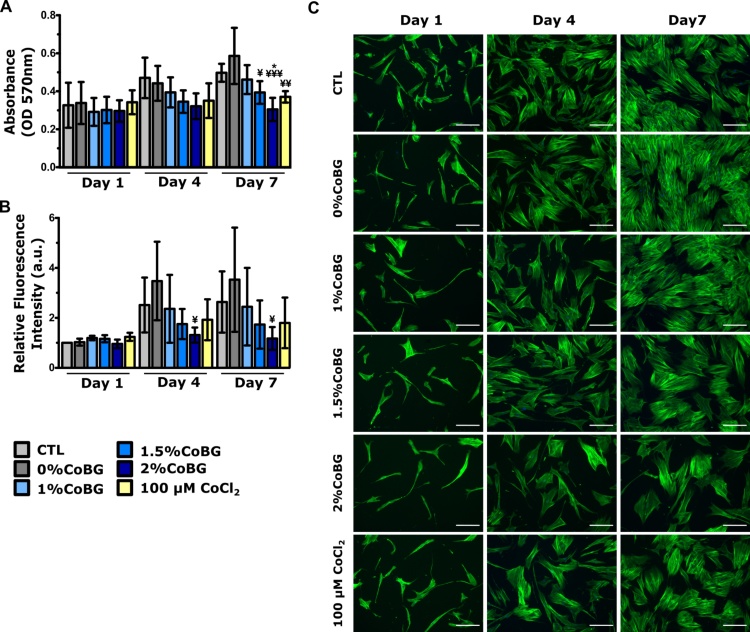
CoBG dose- and time-dependent effect on hMSC metabolic activity and cell abundance. hMSCs were exposed to CoBG-conditioned, CoCl_2_ or control (CTL) medium. After 1, 4 and 7 days, A) MTT assay, B) CyQUANT^®^ Direct Cell Proliferation Assay (both *n* = 3 using different donors) and C) filamentous actin staining were performed. Statistical significance was assessed by two-way ANOVA with Bonferroni *post hoc* test. *p < 0.05 (vs. CTL), ¥*p *< 0.05 (vs. 0%CoBG), ¥¥*p* < 0.01, and ¥¥¥*p *< 0.001. Statistically significant changes over time are not indicated in the figure. A) Metabolic activity was significantly higher in presence of 0%CoBG extracts when days 1 and 7 were compared (*p *< 0.01). B) Comparing days 1 and 4 as well as days 1 and 7, cell number increased significantly for 0%CoBG (*p *< 0.05). Scale bars = 200 μm.

To determine the influence of CoBG dissolution products on cell growth and proliferation, hMSCs were treated with CoBG-conditioned media as well as 100 μM CoCl_2_ for 24 h, 4 and 7 days (Fig. 2). At the indicated time points, hMSC metabolic activity was measured by MTT assay (Fig. 2A). No significant differences in metabolic activity were observed after 24 h and 4 days between conditions. After 7 days of treatment, the metabolic activity of hMSCs cultured in 2%CoBG-conditioned medium was significantly lower than in control and 0%CoBG. Compared to 0%CoBG, the metabolic activity of hMSCs cultured in 1.5%CoBG and 100 μM CoCl_2_ were significantly lower on day 7 indicating a cobalt dose-dependent effect. 0%CoBG tended to enhance hMSC metabolic activity compared to control which is in line with previous findings for human osteoblasts [6]. Overall, metabolic activity increased over time for all conditions except for 2%CoBG which maintained its metabolic activity level from 24 h onwards. As previous studies have shown that exposure to Co^2+^ can influence cell cycle progression and proliferation, it was hypothesised that changes in cell metabolic activity owed to differences in cell proliferation [40], [41]. Cell proliferation was assessed using the CyQUANT^®^ Direct Cell Proliferation Assay and followed a similar trend as metabolic activity (Fig. 2B). Overall, cell number seemed to increase over time for all conditions, yet this was only statistically significant for 0%CoBG (*p <* 0.05). Notably, culture in control and 1%CoBG-conditioned medium resulted in a similar cell proliferation pattern. Concomitantly, cell numbers were similar in the 100 μM CoCl_2_ and 1.5%CoBG condition.

### 3.4 Effect of CoBG dissolution products on hMSC morphology

To assess if cell morphology was altered by the presence of CoBG dissolution products when hMSCs were cultured in 2D, the actin cytoskeleton was stained after 1, 4 and 7 days of exposure (Fig. 2C). hMSCs displayed an elongated, fibroblast-like phenotype after 24 h of culture which progressively changed to a more flattened morphology over time. Overall, there were no differences in cell morphology observed between conditions; however, cell density appeared to be reduced in presence of 1.5%CoBG and 2%CoBG dissolution products after 7 days of culture compared to the other conditions. This observation is in line with the results obtained by MTT and CyQUANT^®^ Direct Cell Proliferation Assay and further indicated a reduction in proliferation rather than apoptosis.

### 3.5 Chondrogenic differentiation in presence of CoBG dissolution products

**Fig. 3:**
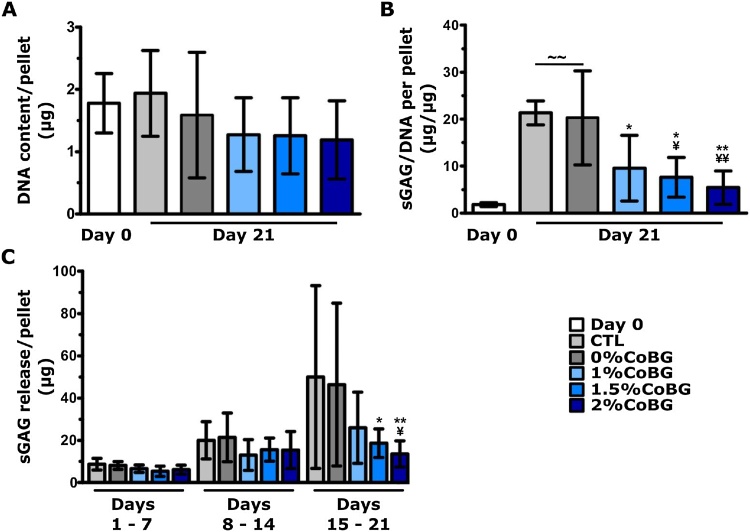
Cobalt dose-dependent reduction of sGAG production and release by hMSCs in pellet culture. hMSC pellets were cultured in CoBG-conditioned or control (CTL) medium for 21 days prior to quantification of A) DNA by Hoechst and B) sulphated glycosaminoglycans (sGAG) by DMMB. A, B) Data are expressed as mean ± SD (day 0, *n* = 3 and day 21, *n* = 5 independent experiments using different donors). C) sGAG release profile of hMSC pellets cultured in CoBG-conditioned medium throughout the culture period. Data are expressed as mean ± SD (*n* = 4 independent experiments using different donors. ∼∼*p* < 0.01 (vs. day 0), *p < 0.05 (vs. CTL), **p < 0.01, ¥*p* < 0.05 (vs. 0%CoBG), ¥¥*p* < 0.01. Statistically significant changes over time are not indicated in the figure. C) The amount of sGAG in retained medium from days 15–21 was significantly higher than in medium from days 1–7 for CTL (*p *< 0.01) and 0%CoBG (*p *< 0.05).

To evaluate whether CoBG mediated HIF-1α stabilisation had an effect on hMSC differentiation towards the chondrogenic lineage, hMSCs were pelleted and cultured in chondrogenic medium in the presence of CoBG dissolution products for 21 days (Fig. 3). The amount of DNA per pellet was reduced in presence of cobalt-doped BGs compared to the control on day 21. Yet, the DNA content of pellets cultured in presence of cobalt containing CoBG dissolution products was not significantly different to day 0 (Fig. 3A). Quantification of sGAG accumulation in pellets normalised to DNA content revealed a cobalt dose-dependent decrease in sGAG production (Fig. 3B). Only hMSC pellets cultured in control or 0%CoBG-conditioned medium contained significantly more sGAG per DNA than pellets on day 0 (*p <* 0.05). In presence of all CoBG dissolution products except for 0%CoBG, the normalised sGAG content was significantly lower when compared to control (*p <* 0.05), and this was particularly pronounced for 2%CoBG (*p <* 0.01). To assess the amount of sGAG that was released rather than accumulated in the pellet matrix, the sGAG content in the medium was measured over the 21 day culture period (Fig. 3C). The release of sGAG from pellets into the medium increased over time for all conditions and followed a similar trend as sGAG accumulation. Yet, this trend was only significant for the control and 0%CoBG conditions when the amount of sGAG in retained medium from days 15–21 was compared to days 1–7. In presence of 0%CoBG dissolution products, pellets released a significantly higher amount of sGAG than in the 1.5%CoBG and 2%CoBG conditions in the final week of culture (days 15–21) further corroborating a cobalt-dose dependent effect.

### 3.6 Characterisation of cartilage-like extracellular matrix formation in presence of CoBG by histology

**Fig. 4:**
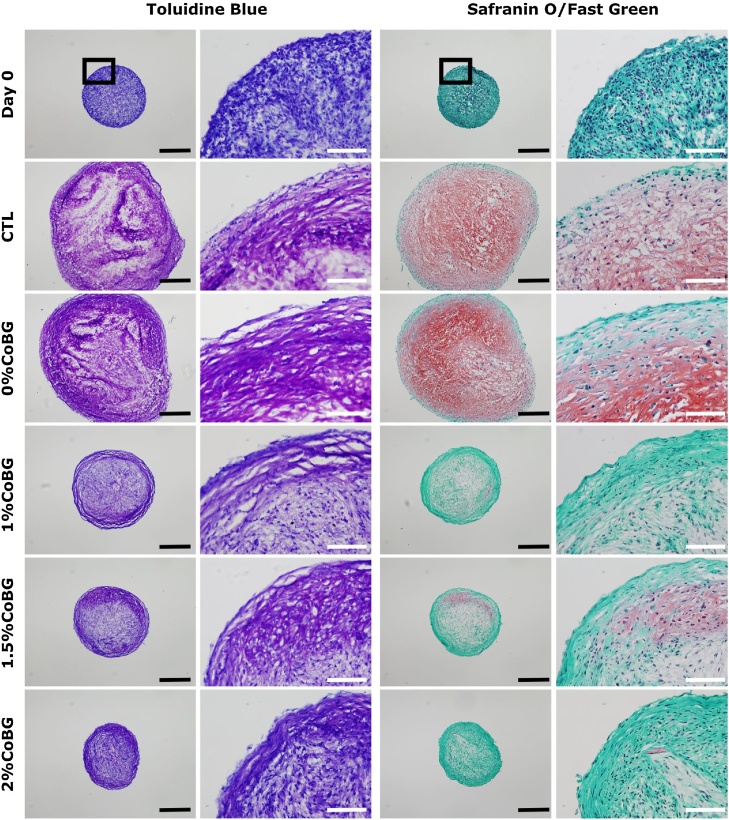
Reduced formation of cartilage-like tissue in the presence of CoBG. Prior to histological assessment, hMSCs were grown as pellets in CoBG-conditioned or control (CTL) medium in chondrogenic conditions for 21 days. Sulphated proteoglycan deposition in the extracellular matrix was assessed by Toluidine Blue (purple metachromasia) and Safranin O staining. Fast Green stains collagens and other proteins. Representative images are shown. The black square marks the area of magnification for the second panel of images for Toluidine Blue and Safranin O/Fast Green staining respectively. Black scale bars = 400 μm, white scale bars = 100 μm. (For interpretation of the references to colour in this figure legend, the reader is referred to the web version of this article.)

Histological analyses were carried out to further assess the quality of the cartilaginous tissue formed by hMSCs in presence of CoBG dissolution products. Histological characterisation by Toluidine Blue and Safranin O staining confirmed the presence of sGAGs throughout the centre of pellets cultured in control and 0%CoBG conditioned-medium demonstrating a good chondrogenic differentiation potential (Fig. 4). However, the cobalt within the CoBG dissolution products appeared to reduce chondrogenic extracellular matrix production indicated by faint or absent Safranin O staining. With increasing cobalt concentration in the conditioned medium, pellets were smaller and appeared denser.

### 3.7 Raman spectral imaging on hMSC pellet sections

**Fig. 5:**
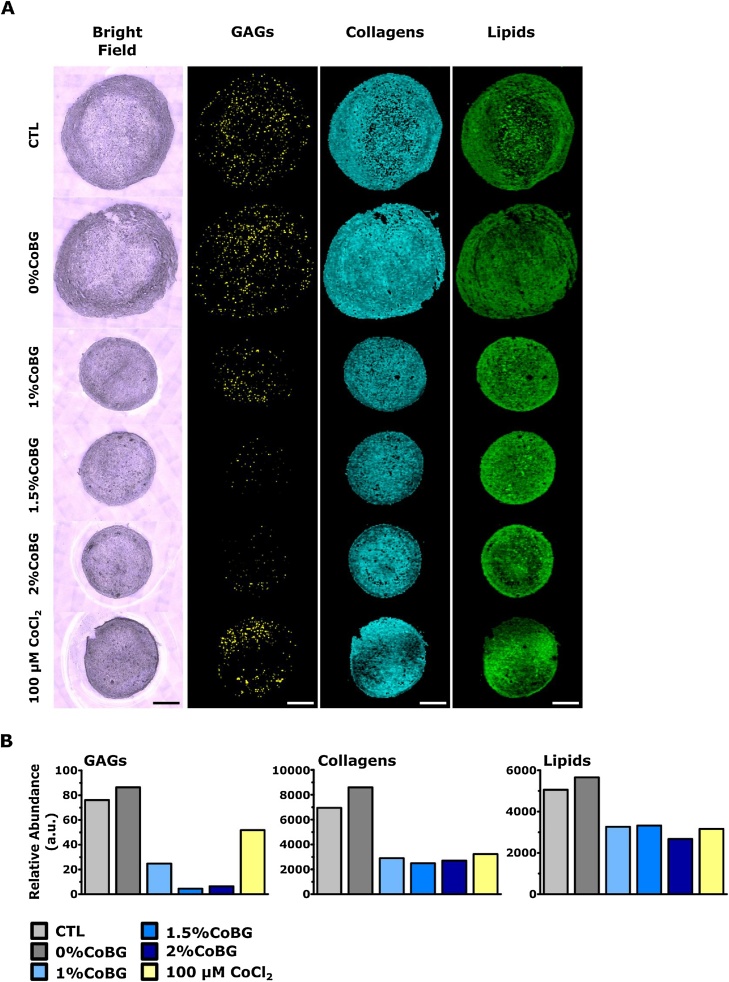
Extracellular matrix characterisation by Raman spectral imaging. Prior to imaging, pelleted hMSCs were exposed to CoBG-conditioned, 100 μM CoCl_2_ or control (CTL) medium in chondrogenic conditions for 21 days. A) Bright field image of respective pellet sections along with pseudo-coloured images of characteristic spectra within the hMSC pellet sections. Scale bars = 300 μm. (B) Semi-quantitative analysis of Raman spectral images of one pellet section per condition. The relative abundance represents the number of pixels within each pellet section positive for the identified discriminator spectra (GAG, collagens or lipids) multiplied by the respective signal intensity.

Raman spectral imaging was performed on hMSC pellet sections to further characterise the tissue (Fig. 5). Raman spectroscopy presents a label-free and non-invasive alternative to conventional techniques which makes it an attractive tool, especially for tissue engineering [1], [42], [43]. Raman spectra provide unique biochemical signatures from each tissue allowing their characterisation and comparison in terms of global biomolecular abundance. Pelleted hMSCs were cultured for 21 days in CoBG-conditioned or control medium, containing chondrogenic supplements. Additionally, control medium was supplemented with 100 μM CoCl_2_ to investigate the effect of cobalt ions in absence of BG extracts. Multivariate analysis of the Raman spectral images highlighted clear differences between the experimental conditions Fig. 5A. MCR clustering analysis identified characteristic spectra that were classified as GAGs, collagens, and lipids. Non-negative least-squares curve fitting analysis provided a semi-quantitative assessment of these discriminators and showed that the GAG, collagen and lipid contents were higher in control and 0%CoBG than in all cobalt-containing conditions. The GAG signal appeared to decrease with increasing cobalt ion concentrations within the BG extracts corresponding to the results obtained by biochemical analysis and histological staining. Addition of CoCl_2_ to chondrogenic medium reduced the GAG content of pellets compared to control and 0%CoBG. While the outcome was similar, the GAG content in CoCl_2_ treated pellets was higher than in the cobalt-containing bioactive glass extract conditions. The signal for collagen and lipids was lower in presence of all cobalt-containing media compared to control and 0%CoBG possibly due to an overall reduction of extracellular matrix production (Fig. 5B). Visual examination of pellets and pellet sections showed that the size of pellets was reduced in presence of Co^2+^ in a concentration-dependent manner.

## 4 Discussion

The incorporation of therapeutic ions into scaffolds is a relatively simple and cost efficient way to promote the bioactivity of biomaterials. BGs are versatile materials and excellent carrier systems for therapeutic ions due to their amorphous structure and partial dissolution in aqueous buffers [1], [3]. In the current study, the effect of cobalt-containing BGs on hMSC chondrogenesis was evaluated. We previously designed a series of hypoxia-mimicking BGs with increasing cobalt contents to evaluate their use for bone tissue engineering applications [7], [38]. We found that the gene expression of hypoxia target genes was up-regulated in CoBG-treated hMSCs. Furthermore, hMSCs produced significantly more VEGF in presence of CoBG extracts [7]. Quinlan et al. demonstrated an increase in VEGF production and tubule formation by HUVECS in presence of CoBG ionic dissolution products [36]. Demonstrating a beneficial effect for bone tissue engineering purposes, the pre-osteoblast cell line MC3T3-E1 seeded on CoBG containing collagen-glycosaminoglycan scaffolds had a higher ALP activity than the cobalt free BG containing collagen-glycosaminoglycan scaffold control [36]. In the current study, we found that short-term exposure to CoBG dissolution products dose-dependently increased HIF-1α abundance in hMSCs which was consistent with the previous study. Furthermore, it was demonstrated that continued exposure to CoBG dissolution products maintained HIF-1α protein at a significantly higher level than in cobalt-free bioactive glass-conditioned medium for at least 48 h. Continuous treatment with CoBG dissolution products or medium supplemented with 100 μM CoCl_2_ reduced hMSC metabolic activity in a time and cobalt dose-dependent manner compared to the control and 0%CoBG conditions. Assessment of cell proliferation indicated that the decrease in metabolic activity was likely due to a lower number of cells in the presence of Co^2+^. Notably, hMSCs cultured in control medium had a similar metabolic activity and proliferation profile to hMSCs exposed to 1%CoBG dissolution products reinforcing the fact that tailoring of the cobalt ion release is crucial. A cobalt dose-dependent reduction of cell proliferation and metabolic activity over time is in line with previous studies [8], [35], [44], [45], [46]. Pacary et al. proposed that this effect was due to a Co^2+^ induced cell cycle arrest by up-regulation of p21 and down-regulation of cyclin D1 rather than apoptosis [41]. In agreement with these findings, no major morphological changes in the presence of CoBG dissolution products were observed indicating that Co^2+^, either within the CoBG dissolution products or as a supplement, did not induce cell death.

To evaluate the impact of cobalt-doped BGs on hMSC chondrogenic lineage commitment, hMSCs were cultured as pellets for up to 21 days in CoBG-conditioned or control medium. As *in vitro* experiments on primary cells have revealed considerable donor variability, 5 independent experiments using hMSCs isolated from 5 different donors were performed. Visual examination of the pellets revealed a reduced pellet size for pellets cultured in Co^2+^-containing medium. Exposure to CoBG dissolution products reduced accumulation and release of sGAG by hMSC pellets in a cobalt concentration-dependent manner. While sGAG production was markedly lower in presence of CoBG dissolution products, histological analysis of the pellets demonstrated the presence of some if little cartilage-like extracellular matrix. Culture of hMSC pellets in 0%CoBG conditioned medium resulted in a similar outcome as the control condition highlighting that cobalt incorporation into the BG composition was likely causative for the reduced cartilage-like matrix production. Raman spectral analysis further corroborated the results obtained by biochemical and histological analysis of pellets. Supplementation of 100 μM CoCl_2_ to the chondrogenic medium resulted in a similar outcome as culture in cobalt-containing BG dissolution products. Overall, incorporation of cobalt in BGs reduced chondrogenic lineage commitment by hMSCs despite HIF-1α stabilisation.

While cobalt ions mimic hypoxia in that they stabilise HIF-1α, there is evidence that cobalt treatment does not fully recapitulate low oxygen tension environments or may indeed induce additional effects in cells such as the induction of apoptosis pathways [23], [40]. Interestingly it has been shown that accumulation of HIF-1α protein in lung epithelial cells decreased after 12 h of exposure to reduced oxygen tension to normal levels while CoCl_2_ treatment maintained a significantly higher concentration at the same time point [47]. Furthermore, Ginouvés et al. demonstrated that HIF-1α degradation is stimulated by increased intracellular oxygen levels in chronic hypoxia and that this mechanism is pivotal for cell survival [48]. Since cobalt ions block HIF-1α degradation independent of oxygen levels, HIF-1α might accumulate throughout the treatment period. Hence, the significantly enhanced HIF-1α abundance after 48 h of exposure to CoBG dissolution products may allude to discrepancies in the mode of action of O_2_ tension and Co^2+^ which may have led to a reduction in chondrogenic differentiation potential. In studies using other biomaterial systems as carriers for Co^2+^, beneficial effects on angiogenesis at concentrations as low as 1.8 μM were observed [49]. Here, 1%CoBG dissolution products did not significantly increase HIF-1α accumulation compared to control but had a substantial effect on hMSC chondrogenesis. This may either suggest that subtle changes in HIF-1α level can have pronounced effects on cell behaviour or that Co^2+^ induced mechanisms independent of the involvement in the HIF-1 pathway that led to a reduction in chondrogenic differentiation. Considering these results, the Co^2+^ concentration in the CoBG-conditioned media prepared for this study might have been too high and/or the exposure time too long. Considering that treatment with 150 μM CoCl_2_ for 24 h has been shown to up-regulate chondrogenic markers in hMSCs, culture in CoBG-conditioned medium for short time periods may be key to exploiting cobalt’s hypoxia-mimicking effect. Alternatively, a CoBG with much lower cobalt incorporation might have resulted in the anticipated positive effect on chondrogenic differentiation.

Recently, several studies suggested that pre-conditioning of MSCs with CoCl_2_ improved their ability to withstand oxidative stress after implantation *in vivo* [50], [51]. Furthermore, there is evidence that hMSCs treated with CoCl_2_ enhanced the engraftment potential of haematopoietic stem cells in a co-culture model. Interestingly, in the conditions used for that study, CoCl_2_ pre-conditioning was shown to outperform reduced oxygen tension [52]. In this context, treatment with 100 μM CoCl_2_ prior to culture in chondrogenic medium enhanced Alcian blue staining and chondrogenic marker gene expression in a murine MSC cell model system [53]. Considering these data, shorter exposure times to cobalt containing dissolution products may prime cells to withstand oxidative stress and/or prepare them to differentiate towards the chondrogenic lineage.

## 5 Conclusions

This study demonstrates that cobalt incorporation into BGs dose-dependently reduced chondrogenic differentiation of hMSCs despite HIF-1α stabilisation. In the presence of 0%CoBG dissolution products cell proliferation seemed enhanced and commitment to the chondrogenic lineage was supported identifying BG as a suitable (bone) substrate for osteochondral scaffolds. The results, strengthened by the use of 5 different donors, thus highlight that both the concentration and the exposure time to cobalt containing BG dissolution products influence the hMSC response. Hence, the extent of cobalt incorporation into biomaterials needs to be carefully evaluated and tailored to the application.

## Author contributions

Conceptualisation: EL, HA, MMS; Methodology: EL, HA; Formal Analysis: EL, HA, CK; Investigation: EL, AKS, CK; Resources: JRJ, MA, MMS; Writing − Original draft: EL, HA; Writing − Review & Editing: EL, HA, MP, JRJ, MMS; Supervision: HA, MP, MA, MMS

## Funding

The work was supported by the European Commission funding under the 7th Framework Programme (Marie Curie Initial Training Networks; grant number: 289958, Bioceramics for bone repair). M.M.S. and H.A were financially supported by the Medical Engineering Solutions in Osteoarthritis Centre of Excellence funded by the Wellcome Trust and the Engineering and Physical Sciences Research Council (088844). H.A. was also partially funded by the Value In People award from the Wellcome Trust Institutional Strategic Support Fund (097816/Z/11/B). The funders had no role in study design, data collection and analysis, decision to publish, or preparation of the manuscript. The raw data supporting this paper is available upon request from m.stevens@imperial.ac.uk.

## Competing interests

M.M.S and J. R. J are co-inventors on patents related to bioactive glasses.

## Glossary

BG: Bioactive glasses
CoBG: Cobalt-containing bioactive glasses
HIF-1: Hypoxia inducing factor-1
hMSC: Human mesenchymal stem cell
sGAG: Sulphated glycosaminoglycan
