# Supplementary material for: Cobalt-containing bioactive glasses reduce human mesenchymal stem cell chondrogenic differentiation despite HIF-1α stabilisation
Source: J Eur Ceram Soc. 2018 Mar;38(3):877–86. doi: 10.1016/j.jeurceramsoc.2017.08.001 (PMC5738970; doi:10.1016/j.jeurceramsoc.2017.08.001)
Supplement: Supplementary file 1 [file mmc1.docx]

**Supplements**

**Supplement 1: Tailoring of bioactive glass compositions allowed controlled release of cobalt ions into α-MEM medium.** Elemental concentrations of CoBG ionic dissolution products (Si, Ca, P and Co) in α-MEM medium. CoBG particles were incubated in α-MEM for 4 h under constant agitation. Elemental concentrations of CoBG-conditioned and α-MEM medium (*n* = 7) as well as α-MEM medium supplemented with 100 µM CoCl_2_ (*n* = 3) were measured by ICP-OES. Ion concentrations are presented as mean ± SD of 7 or 3 independent experiments, respectively (in μg/mL unless otherwise indicated).

|  | **Si** | **Ca** | **P** | **Co** | **Co (µM)** |
| --- | --- | --- | --- | --- | --- |
| **α-MEM** | **ND** | **65 ± 4** | **29 ± 5** | **ND** | **/** |
| **0%CoBG** | **66 ± 4** | **158 ± 9** | **15 ± 3** | **ND** | **/** |
| **1%CoBG** | **62 ± 5** | **148 ± 10** | **18 ± 3** | **6 ± 1** | **105 ± 15** |
| **1.5%CoBG** | **64 ± 5** | **158 ± 9** | **21 ± 4** | **11 ± 1** | **181 ± 14** |
| **2%CoBG** | **62 ± 3** | **156 ± 14** | **17 ± 3** | **16 ± 2** | **276 ± 29** |
| **100 µM CoCl_2_** | **ND** | **59 ± 2** | **29 ± 2** | **5 ± 1** | **84 ± 12** |
